# Fluid accumulation post cardiac surgery and risk for renal replacement therapy

**DOI:** 10.1186/cc13578

**Published:** 2014-03-17

**Authors:** EM Moore, A Tobin, D Reid, J Santamaria, R Bellomo

**Affiliations:** 1 Monash University, Melbourne, Australia; 2 St Vincent's Hospital, Melbourne, Australia

## Introduction

We assessed the impact of fluid accumulation on the development of acute kidney injury (AKI) and need for continuous renal replacement therapy in cardiac surgical patients. Fluid accumulation has been associated with negative outcomes including development of AKI in critically ill patients [1-3]. As cardiac surgical patients commonly receive large volumes of i.v. fluid within 24 hours of surgery, they could be at risk of the harmful effects of fluid accumulation.

## Methods

We performed a retrospective analysis of prospectively collected data on all patients admitted after cardiac surgery to St Vincent's Hospital ICU, Melbourne, Australia from 1 July 2004 to 30 June 2012 (*n *= 3,207). The fluid accumulation percentage (total urine and chest drain losses subtracted from total i.v. intake (l) /weight (kg) × 100) was calculated for 18 hours post surgery as most patients were in the ICU for this period. Acute Kidney Injury Network (AKIN) creatinine criteria were used to classify AKI using creatinine adjusted for fluid balance.

## Results

Renal replacement therapy was performed on 136 patients in this group (4.2%). The fluid accumulation percentage was associated with an 8% increase in odds for AKI (OR (Cl), 1.08 (1.04 to 1.12)), and a 13% increase in odds for requiring renal replacement therapy (1.13 (1.05 to 1.21)) for each percent increase in fluid accumulation (l/kg%) after cardiac surgery, after adjusting for variables including APACHE score, cardiac failure, type of surgery, and inotrope use in multivariate analysis.

## Conclusion

In this relatively homogeneous patient group undergoing cardiac surgery, postoperative percent fluid accumulation at 18 hours was associated with AKI and need for renal replacement therapy. Whether there is residual confounding due to indication for fluid use or unmeasured risk factors requires further investigation in controlled trials.
